# Measurement of bio-physical signals for posture movement on the transformation system

**DOI:** 10.1080/13102818.2014.949042

**Published:** 2014-10-22

**Authors:** Jeong-lae Kim, Kyu-dong Kim

**Affiliations:** ^a^ Department of Biomedical Engineering, Eulji University, Seoul, Korea; ^b^ Department of Medical IT and Marketing, Eulji University, Seoul, Korea

**Keywords:** posture movement, vision, vestibular, somatosensory apparatus, CNS

## Abstract

A signal transformation system of posture movement for the stable state was designed in order to measure the bio-physical signal. To estimate the subject in a stable state on the basis of the bio-physical signal in the posture movement, the conditions of vision, vestibular, somatosensory apparatus and the central nervous system (CNS) were detected. Based on the vision condition, there was a greater average variation (Vi-α_AVG-MAX_ and Vi-α_AVG-MIN_) in position with eyes closed and eyes opened (PC and PO), which was 27.11 ± 6.36 units. The vestibular condition showed a slightly larger average variation (Ve-α_AVG-MAX_ and Ve-α_AVG-MIN_) in the body position with PC and PO, which was 6.60 ± 1.65 units. The somatosensory condition demonstrated a lower average variation (So-α_AVG-MAX_ and So-α_AVG-MIN_) in position with PC and PO that amounted to 3.653 ± 2.424 units. The CNS condition was confirmed to indicate very little average variation (C-α_AVG-MAX_ and C-α_AVG-MIN_) in body position with PC and PO that was at 0.401 ± 0.56 units. As the model depends on the bio-physical transformation system of posture movement, the average values of these perturbations were computed (0.01–2 Hz, range of Fourier frequency). The system consists of a data algorithm, an acquisition system, a data signal processing unit and a network system for the evaluated stability.

## Introduction

The effect of bio-physical signals for human posture is enormous, both for individuals suffering from balance impairment and for the society at large. One major concern includes postural instability, which is associated with considerable attack rate and frequency of falls. As a consequence, the quality of life of patients with balance impairment and falls is remarkably low.[1] The management of postural instability in patients is hindered by the intricate estimation of balance. The current tendency in practice is a combination of history taking and physical exercise, but also needs other variable conditions.[2]

Posture reflex techniques are used to search the active and passive rule of balance under a diversity of conditions. The fundamental elements of most posture reflex techniques include the ability for posture or balance to be unaffected, and evaluate the response of the subject to such interactions. Available posture reflex techniques have recently been widely reviewed.[3] Balance can be disturbed when the support surface makes an up-and-down motion, horizontal and vertical displacements.[3] Most techniques use rapid and brief perturbations in order to study the immediate postural reactions, but slow and fast movements have been used to investigate the adaptation to movement and postural control mechanisms.[4]

A network access protocol has been proposed for real-time transformation systems. The message in a real-time transformation system is associated with a timing restriction, generally in the form of a mode.[5] The most commonly used network in distributed real-time systems is the network access protocol.[6]

In this study, a bio-physical signal transformation system was developed to estimate the physical signal in order to detect posture movement, especially the parameters of human condition included in the opening and closing of an eye. This system was used to prove the transformation capability through the acquisition system to obtain local data in the near area.

## Materials and methods

### Subjects

The experiments were conducted using the database of Eulji University (Seoul, Korea), which is collected from 19 individuals (including 15 males and 4 females, Asian race). The age of the participants was between 19 and 30 years. In terms of occupation, they were all university students at our school. Informed consent for participation in the study was obtained from all participants.

### Components of the bio-physical signal system

**Figure 1.**
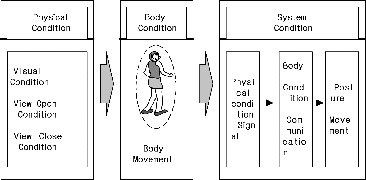
Structure of the bio-physical signal system for postural movement.

Bio-physical compound data are collected from signals for posture movement based on the studied conditions (Figure 1). The bio-physical parameter was a posture movement for balance during visual condition of the ‘view open’ and ‘view close’ condition. Data analysis gave an account of a physical condition signal for system condition (Figure 1). Data analysis was achieved through frequency.[7]

As the systems are extremely complicated to be understood in detail and to be tested, simplified models are constructed.[8] The system for signal component achieves complete automation of the testing process.[9]

Since the time-labelled bio-physical signal system is infinite due to the infinitely large number of visual condition transitions, we can deal with it to generate view open/close cases. The challenge is therefore to considerably reduce the states of body movement in the system. To achieve this, a body movement of equivalent fluctuation is defined on the basis of the physical signal in order to acquire physical states equivalent to the body condition. The resulting time-labelled bio-physical signal system is called confined spaces.[10,11]

## Results and discussion

**Figure 2.**
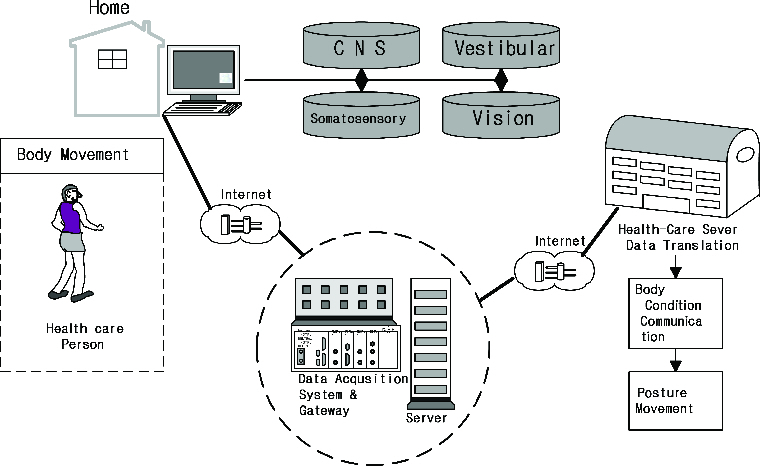
Proposed bio-physical signal transformation system for postural movement.

The proposed bio-physical signal system aims to check the movement of the posture for monitoring a subject in the health care service centre, i.e. the body movement (Figure 2). This system can function normally both when the body condition is stable and restful and when it is abnormal and unstable.[12–15]

### Background

Many posturography studies investigated the effects of specific diagnosis modes on balance and postural control. A mode that received considerable attention is the possible effect of posture movement on signal control in different types of posturography.[16] The effect of exercise has to be investigated with the posture movement in posturography. In posturography, balance exercise improved the performance on the posture movement.[17] Posture sway during normal conditions was correlated with the frequency of perturbations while doing exercise. Moreover, self-reported movement was consistently correlated with the swaying frequency in more parameter.[18] Even healthy subjects show substantial variability in their postural responses upon postural movement.[19]

### Algorithm of the bio-physical signal system

**Figure 3.**
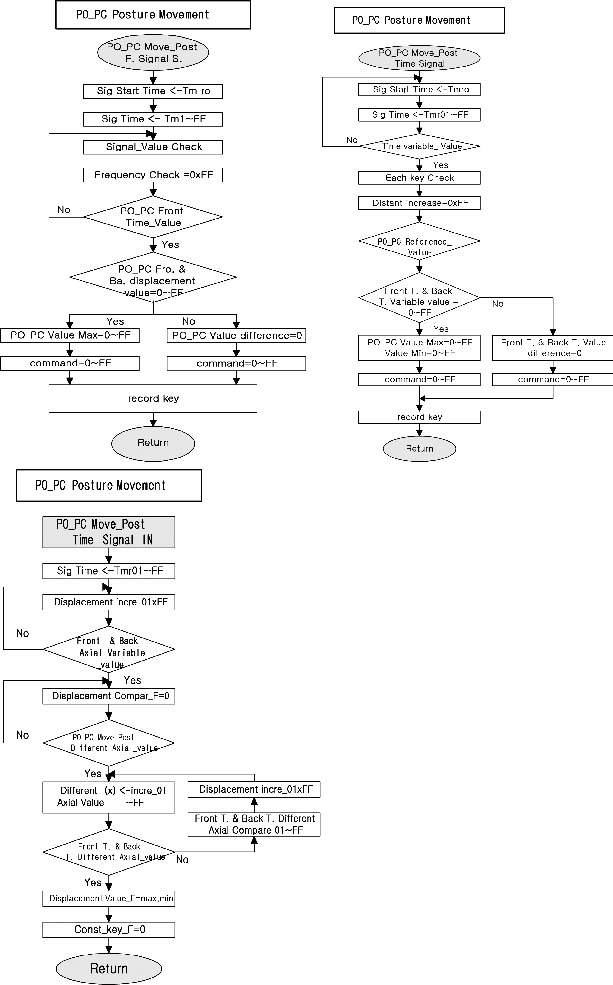
Block diagram of the proposed bio-physical signal transformation system for postural movement.

The posture movement aspects to be specified for a signal process are similar to those in the specification of the rectified posture (Figure 3).[20–22] The specification of a signal process included the definition of the inputs to be applied to the posture with eyes open and eyes closed (PO_PC) signal possibly in response to particular outputs received from the PO_PC value.

Several software algorithms are used in modules communicating PO_PC posture movement. We addressed the item of bio-physical signal systems in several time models. The following is a list of issues related to distributed testing of communication algorithms: (1) first, the frequency of the front and back signal for posture movement is defined. (2) There follows the time variable of the front and back time point. (3) Then, the axial sway displacement of front and back time is presented by the reference for PC and PO.

The frequency of the front and back signal in a transition from Front_time_value to Fro-Ba displacement gives ‘Yes’ as an output action, and a value ‘Max’ condition, while the transition from Fro-Ba displacement to Val-difference can execute on input ‘No’ at any time, and resets to zero command value. A distinction between a posture movement signal case and a front-back displacement case is made. The most important aspect is the form of the connection through which the frequency case interacts with the PO_PC value.

The time variable of the front and back time point in a transition from Reference_value to Front-Back_T variable value has ‘Yes’ as an output condition, and a value ‘Max–Min’ condition, while the transition from Front-Back_T variable value to T-variable difference can execute on input ‘No’ at any time, and resets to zero command value. A clear distinction between a posture movement time signal case and a reference displacement case is made. The most important aspect is the form of the connection through which the frequency case interacts with the PO_PC value of posture movement.

The axial sway displacement of front and back time by the reference in a transition from Front-Back Axial variable value to Move-Post Diff-Axial variable value has ‘Yes’ as a flow action; a transition from Move-Post Diff-Axial variable value to Front-Back_T_different Axial value has ‘Yes’ as a flow action, and a value ‘Displacement’ condition; while the transition from Front-Back_T_different Axial value to T_different Axial comparison can execute on input ‘No’ at any time, and reconstruct to zero Const-key value. A sharp distinction between a front_back signal case and an axial displacement case is made. The most important aspect is the form of the connection through which the axial displacement case interacts with the PO_PC value of sway.

### Experimental results and analysis

The average results from the analysis of the studied parameters and conditions in the 19 participants are shown in Table 1.

**Table 1.** Average of posture movement difference measures to the various Vision (Vi-α_Avg_), Vestibular (Ve-α_Avg_), Somatosensory (So-α_Avg_) and CNS (C-α_Avg_) conditions.

Average α	Vi-α_Avg_	Ve-α_Avg_	So-α_Avg_	C-α_Avg_
α_MAX-POPC_	30.63 ± 7.78	10.88 ± 3.36	4.86 ± 2.438	0.688 ± 0.57
α_MIN-POPC_	3.52 ± 1.42	4.28 ± 1.71	1.217 ± 0.214	0.287 ± 0.004
α_AVG-POPC_	12.47 ± 4.23	6.78 ± 1.69	2.32 ± 0.93	0.386 ± 0.141

Note: Average of α_MAX-POPC_, α_MIN-POPC_ and α_AVG-POPC_.

#### Comparison of α_MAX-POPC_, α_MIN-POPC_ and α_AVG-POPC_

**Figure 4.**
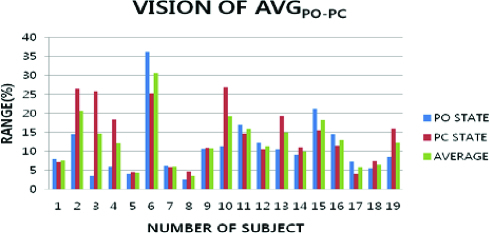
Average data of vision condition and the postural movement in PO and PC.

The vision condition was verified as a variation for the Vi-α_AVG-MAX_ and Vi-α_AVG-MIN_ (Figure 4). The greater average difference between Vi-α_AVG-MAX_ and Vi-α_AVG-MIN_ was with PC and PO of body. The greater average difference between Vi-α_AVG-MAX_ and Vi-α_AVG-MIN_ was at 27.11 ± 6.36 units. Vi-α_AVG-AVG_ was presented slightly greater at 12.47 ± 4.23 units. Vi-α_MAX-POPC_ was at 30.63 ± 7.78 units, which varied to a greater extent than Vi-α_AVG-AVG_. Vi-α_MIN-POPC_ was 3.52 ± 1.42 units, which varied a more than Vi-α_AVG-AVG_.

The vision condition was observed to vary more with posture movement of PO and PC. The vision average showed larger fluctuation at 7.17 ± 3.55 units with Vi-α_AVG-POPC_. Therefore, the parameter that the body sway influences is the vision. This indicated that the vision condition was more important for postural control when the visual characteristics showed the largest variance.

**Figure 5.**
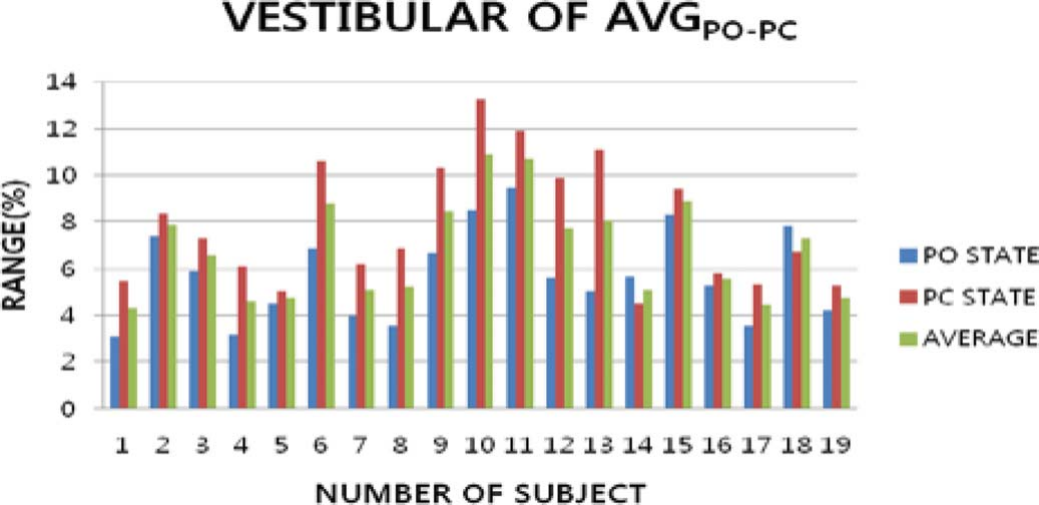
Average data of vestibular condition and the postural movement in PO and PC.

The vestibular condition was determined based on the variation of Ve-α_AVG-MAX_ and Ve-α_AVG-MIN_ (Figure 5). A little larger average difference between Ve-α_AVG-MAX_ and Ve-α_AVG-MIN_ was observed with PC and PO of body. The larger average difference between Ve-α_AVG-MAX_ and Ve-α_AVG-MIN_ was at 6.60 ± 1.65 units. Ve-α_AVG-AVG_ was slightly larger at 6.78 ± 1.69 units. Ve-α_MAX-POPC_ was at 10.88 ± 3.36 units, which varied slightly more than Ve-α_AVG-AVG_. Ve-α_MIN-POPC_ was at 4.28 ± 1.71 units, which varied a little more than Ve-α_AVG-AVG_.

The vestibular condition showed a slightly larger variation with posture movement of PO and PC. The vestibular averages showed slightly larger variation at 4.10 ± 1.67 units with Ve-α_AVG-POPC_. The vestibular condition was less influenced by the posture movement during body sway. This showed that the vestibular condition was more important for postural control when the vestibular characteristic presented the largest variance.

**Figure 6.**
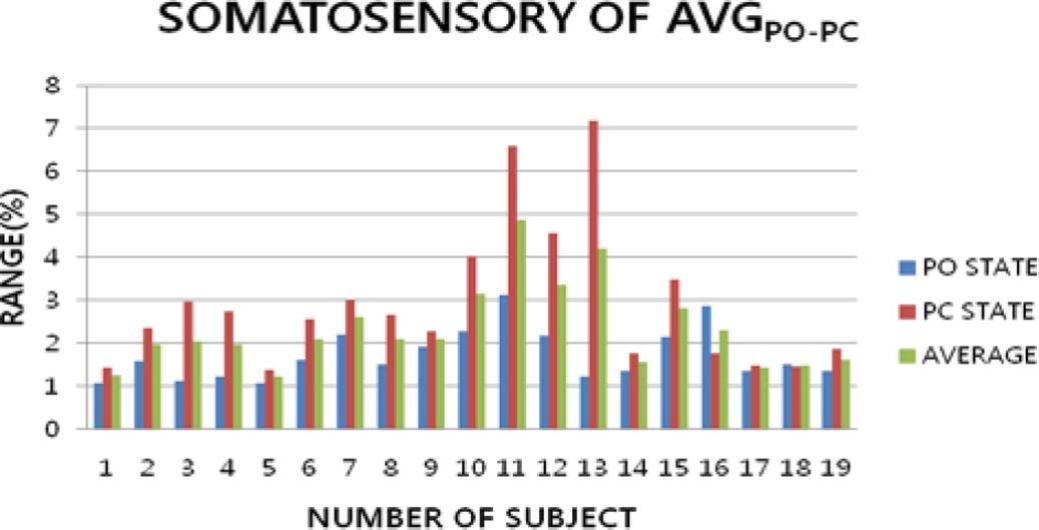
Average data of somatosensory condition and the postural movement in PO and PC.

The somatosensory condition showed a variation for the So-α_AVG-MAX_ and So-α_AVG-MIN_ (Figure 6). The smaller average difference between So-α_AVG-MAX_ and So-α_AVG-MIN_ was with PC and PO of body. The smallest average difference between So-α_AVG-MAX_ and So-α_AVG-MIN_ was at 3.653 ± 2.424 units. So-α_AVG-AVG_ was observed to be slightly smaller at 2.32±0.93 units. So-α_MAX-POPC_ was at 4.86 ± 2.438 units, which varied slightly less than So-α_AVG-AVG_. So-α_MIN-POPC_ was at 1.217 ± 0.214 units, which varied a little less than So-α_AVG-AVG_.

The somatosensory condition showed smaller variation with posture movement of PO and PC. The somatosensory average was shown to vary only a little at 2.54 ± 1.50 units with So-α_AVG-POPC_. Therefore, the somatosensory condition was less influenced by posture movement. This showed that the somatosensory condition was more important for postural control when the somatosensory characteristic demonstrated greater variation.

**Figure 7.**
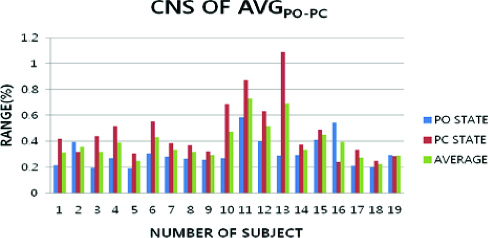
Average data of CNS condition and the postural movement in PO and PC.

The central nervous system (CNS) condition was confirmed to vary for the C-α_AVG-MAX_ and C-α_AVG-MIN_ (Figure 7). The smallest average difference between C-α_AVG-MAX_ and C-α_AVG-MIN_ was with PC and PO. The smallest average difference between C-α_AVG-MAX_ and C-α_AVG-MIN_ was at 0.401 ± 0.56 units. C-α_AVG-AVG_ was slightly larger at 0.386 ± 0.141 units. C-α_MAX-POPC_ was at 0.688 ± 0.57 units and varied a little more than C-α_AVG-AVG_. C-α_MIN-POPC_ was at 0.287 ± 0.0004 units and varied a little less than C-α_AVG-AVG_.

The CNS condition was observed to vary less with posture movement of PO and PC. The CNS average was shown to be a little less at 0.302 ± 0.43 units with C-α_AVG-POPC_. Thus, CNS condition was little affected by posture movement during body sway. This showed that the CNS condition was more effective for postural control when the CNS characteristic presented a smaller variance.

### Performance evaluations

**Figure 8.**
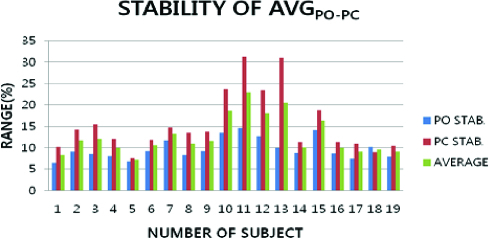
Signal data of stability by the normal postural movement in PO and PC (Stab-_APM_, Avg_MAX-POPC_, Avg_MIN-POPC_, Avg_AVG-POPC_).

The signal data to estimate bio-physical signal results of the stable state detected on the basis of signal in the posture movement are shown in Figure 8. This condition was verified with a little greater average variation (Avg_AVG-POPC_) with a stability of body that was at 20.63 ± 12.42 units.

The average difference of postural movement was illustrated by the values of Stab-_APM_ (including Avg_AVG-POPC_). The results are shown in Table 2. To present a perturbation, the average difference was larger in the PC condition. The stability part showed a little more stable state in the PO condition.

**Table 2.** Average of posture movement difference measures to the various Stab-_APM_ values.

Average	Avg_MAX-POPC_	Avg_MIN-POPC_	Avg_AVG-POPC_
Stab-_APM_	22.926 ± 11.715	7.119 ± 0.444	20.63 ± 12.42

Note: Average of Avg_MAX-POPC_, Avg_MIN-POPC_ and Avg_AVG-POPC_.

## Conclusions

In this paper, we propose a model design of a signal transformation system of postural movement for the stable state. The bio-physical signals for the subject at the stable state were estimated in order to achieve detection of the postural movement on the basis of the signal. The detected conditions were Vision, Vestibular, Somatosensory and CNS. Further studies will focus on the possibility for the proposed system to be used for correction of posture.
